# Routinely Derived Metabolic Indices for IDF‐Defined Metabolic Syndrome in Chinese Adults: A Cross‐Sectional Study

**DOI:** 10.1155/jdr/4648598

**Published:** 2026-10-16

**Authors:** Qiuman Lin, Fen Zhou, Yongjian Zhang, Qianqian Zhong, Jianxin Liu

**Affiliations:** ^1^ Department of Physical Examination Peking University Shenzhen Hospital Shenzhen China pkuszh.com

**Keywords:** central adiposity, cross-sectional study, health examination, metabolic syndrome, receiver operating characteristic curve, triglyceride-glucose index

## Abstract

**Objective:**

The aim of this study is to compare the standardized associations, cross‐sectional discrimination, and distributional robustness of 13 routinely derived metabolic indices for International Diabetes Federation (IDF)‐defined metabolic syndrome (MetS).

**Methods:**

We included 9660 Chinese adults who underwent routine health examinations at Peking University Shenzhen Hospital between August 2024 and March 2026. Associations were assessed using logistic regression with standardized indices and restricted cubic splines, and overall and prespecified subgroup discrimination were evaluated using receiver operating characteristic analyses. Fully adjusted analyses included age, sex, low‐density lipoprotein cholesterol, total bilirubin, blood urea nitrogen, creatinine, and uric acid. Robustness was examined after 1st–99th percentile winsorization using self‐standard deviation (self‐SD) and fixed main‐sample standard deviation (fixed main‐SD) standardization.

**Results:**

Among 9660 participants, 1635 had MetS (16.93%). The cardiometabolic index (CMI) showed the strongest standardized association (odds ratio (OR) per standard deviation increment, 19.437), followed by the triglyceride‐glucose waist circumference index (TyG‐WC; OR, 16.332). TyG‐WC had the highest overall area under the receiver operating characteristic curve (AUC, 0.934; 95% confidence interval (CI), 0.929–0.939) and the highest AUC in 9 of 11 prespecified strata. After winsorization, TyG‐WC retained strong standardized associations under self‐SD and fixed main‐SD scaling (ORs, 17.181 and 18.638, respectively) and stable discrimination (AUC, 0.935), whereas CMI, the lipid accumulation product (LAP), visceral adiposity index (VAI), and triglyceride‐total cholesterol‐body weight index (TCBI) showed substantial scale dependence. All indices showed nonlinear associations (*p* < 0.001).

**Conclusion:**

The 13 indices differed in association strength, discrimination, and distributional robustness. TyG‐WC showed the most balanced overall profile and maintained strong discrimination across most prespecified subgroups. These findings support the further evaluation of TyG‐WC in routine health examination settings. Further validation in independent multicenter populations is required.

## 1. Introduction

Metabolic syndrome (MetS) is characterized by the clustering of central obesity, elevated blood pressure, dysglycemia, and atherogenic dyslipidemia, reflecting an interrelated metabolic phenotype rather than a single abnormality [1–3]. Routine health examinations commonly provide waist circumference, blood pressure, fasting plasma glucose, and lipid measurements, which capture complementary aspects of glycemic‐lipid metabolism, adiposity, and insulin resistance‐related status and provide the basis for deriving and comparing a range of routine metabolic indices.

Among these routinely derived measures, the triglyceride‐glucose index (TyG), TyG‐waist circumference (TyG‐WC), and TyG‐waist‐to‐height ratio (TyG‐WHtR) combine glycemic lipid information with central‐adiposity measures [4–6]. The lipid accumulation product (LAP), visceral adiposity index (VAI), and cardiometabolic index (CMI) characterize metabolic abnormalities through lipid accumulation‐, visceral adiposity‐, and triglyceride/high‐density lipoprotein cholesterol (TG/HDL‐C)‐related features [7–10]. The metabolic score for insulin resistance (METS‐IR) provides a noninsulin‐based surrogate for insulin resistance [11], whereas the Chinese visceral adiposity index (CVAI) emphasizes visceral adiposity‐related characteristics in Chinese populations [12, 13]. In addition, the cholesterol‐HDL‐glucose index (CHG) integrates total cholesterol, HDL‐C, and fasting glucose [14–16]; the triglyceride‐total cholesterol‐body weight index (TCBI) reflects a composite lipid‐weight burden [17, 18]; and the non‐HDL‐C to HDL‐C ratio (NHHR), together with its waist‐modified form, NHHR‐WC, captures atherogenic lipoprotein balance with or without additional central‐adiposity information [19, 20].

For comparative interpretation, these 13 indices can be broadly organized into three groups according to their dominant components and metabolic emphasis: glycemic‐lipid/insulin‐resistance‐related indices, comprising TyG, TyG‐WC, TyG‐WHtR, CHG, CHG‐WC, and METS‐IR; central adiposity/lipid accumulation‐related indices, including CMI, LAP, VAI, and CVAI; and lipid burden/lipoprotein balance‐related indices, including TCBI, NHHR, and NHHR‐WC. Although these measures overlap in their constituent variables and metabolic information, their dominant constructs differ, and they may, therefore, show distinct patterns of association with and discrimination of MetS.

However, head‐to‐head evidence remains limited. Few studies have simultaneously compared associations with MetS and discrimination of MetS defined by the International Diabetes Federation (IDF) across these indices within the same population and adjustment framework, while also examining subgroup performance, nonlinear associations, and distributional handling. Therefore, we systematically compared 13 routinely derived metabolic indices among Chinese adults undergoing health examinations, focusing on their standardized associations with and cross‐sectional discrimination of IDF‐defined MetS, with subgroup, restricted cubic spline, and robustness analyses used to evaluate the consistency and stability of performance.

## 2. Methods

### 2.1. Study Design and Participants

This single‐center cross‐sectional study analyzed the routine health examination records of adults attending the Department of Physical Examination, Peking University Shenzhen Hospital, between August 2024 and March 2026. A prespecified participant‐level cleaning sequence verified the subject identifiers and medical history, retained one examination per participant, and applied the age, plausibility, and completeness criteria. The final analytic sample comprised 9660 adults.

Adults aged at least 20 years were eligible if sex, anthropometric measures, blood pressure, fasting plasma glucose level, and required lipid measurements were complete and within prespecified plausible ranges. We excluded participants with a self‐reported history of cardiometabolic or major cardiovascular disease/intervention, thyroid disease or surgery, chronic respiratory or neurologic/neuromuscular disease, malignancy, major surgery, or treatment likely to materially alter glucose, lipid, blood pressure, or body composition, as recorded during the health history assessment. Any such history in a record linked to the same subject identifier triggered participant‐level exclusion. For repeated examinations among otherwise eligible participants, the most complete record was retained or the most recent within the study window when completeness was comparable.

The Research Ethics Committee of Peking University Shenzhen Hospital approved the study (Peking University Shenzhen Hospital Ethics Review [Research] [2026] No. 102) and waived the requirement for informed consent because this retrospective analysis used existing routine health examination records and involved no additional interventions. This study adhered to the principles of the Declaration of Helsinki and followed the approved institutional protocol.

### 2.2. MetS Definition

MetS was defined according to adult IDF criteria [2, 3]. For Chinese adults, central obesity was defined as waist circumference ≥ 90 cm in men or ≥ 80 cm in women. With central obesity present, MetS required at least two of the following: triglycerides ≥ 1.70 mmol/L; HDL‐C < 1.03 mmol/L in men or < 1.29 mmol/L in women; systolic blood pressure ≥ 130 mmHg or diastolic blood pressure ≥ 85 mmHg; or fasting plasma glucose ≥ 5.6 mmol/L. Since participants with such self‐reported histories were excluded before the analysis, the classification relied on measured examination components rather than prior diagnoses or medication use.

### 2.3. Exposure Indices and Comparative Grouping

We evaluated 13 indices: TyG, TyG‐WC, TyG‐WHtR, CHG, CHG‐WC, TCBI, CMI, LAP, NHHR, NHHR‐WC, METS‐IR, VAI, and CVAI, organized into the same three broad groups described above. The complete formulae and input‐unit conventions are provided in Table S1. Published formulae using conventional laboratory units were retained with explicit internal conversion from mmol/L. Sex‐specific equations were applied for LAP, VAI, and CVAI; the prespecified minimum waist rule for LAP is also specified in Table S1.

### 2.4. Covariates

Model 1 was adjusted for age and sex. Model 2 additionally included low‐density lipoprotein cholesterol, total bilirubin, blood urea nitrogen, creatinine, and uric acid levels. These covariates were selected a priori and applied consistently across index‐specific models. Model 2 was the primary adjusted model.

### 2.5. Statistical Analysis

Continuous variables are summarized as mean (standard deviation) or median (interquartile range) and categorical variables as *n* (%). Between‐group comparisons were performed using Student′s *t*‐test, Wilcoxon rank‐sum test, chi‐squared test, or Fisher′s exact test, as appropriate. Each index was standardized before logistic regression analysis, with odds ratios (ORs) and 95% confidence intervals (CIs) reported per standard deviation increment. Receiver operating characteristic (ROC) analysis was used to assess cross‐sectional discrimination; adjusted ROC curves were based on model‐predicted probabilities, and the area under the curve (AUC) was used to summarize performance. The Youden index was used to identify sample‐specific operating points. In the adjusted ROC models, these were first identified on the predicted probability scale. For Model 2 analyses, the corresponding index values were then derived under a reference covariate profile, with continuous covariates fixed at their medians and categorical covariates at their modal levels within the relevant analytic sample or stratum. These operating references were interpreted as sample‐specific screening references rather than clinically validated or universal diagnostic thresholds, because they depend on case mix, outcome definition, and adjustment set.

Restricted cubic splines with four knots were used to examine nonlinear associations. Model 2 ROC analyses were repeated by age group (20–44, 45–64, and ≥ 65 years), sex, and combined age‐by‐sex strata; age and/or sex were omitted when constant within a stratum, with the remaining Model 2 covariates retained. These prespecified subgroup analyses were descriptive. Variance inflation factors (VIFs) were used to assess multicollinearity. Sensitivity analyses were performed by winsorizing the selected underlying continuous variables at the 1st and 99th percentiles, recalculating all indices, and repeating the main logistic models. For these winsorized logistic analyses, per‐SD estimates were calculated in parallel using standard deviations recalculated within the winsorized dataset (self‐SD) and the corresponding standard deviations from the main analysis dataset (fixed main‐SD). Skewness before and after winsorization was summarized as a descriptive measure of distributional change. To examine whether distributional handling affected discrimination, the main Model 2 ROC analysis was repeated using the recalculated winsorized indices. No participants were removed; all required variables were complete, and all tests were two‐sided.

## 3. Results

### 3.1. Baseline Characteristics

The final analytic sample comprised 9660 adults, of whom 1635 met the IDF definition of MetS (16.93%). Central obesity, hyperglycemia, elevated blood pressure, elevated triglycerides, and low HDL‐C levels were present in 45.42%, 10.48%, 30.40%, 25.83%, and 17.45% of the participants, respectively. The participant selection process is illustrated in Figure 1.

**Figure 1:**
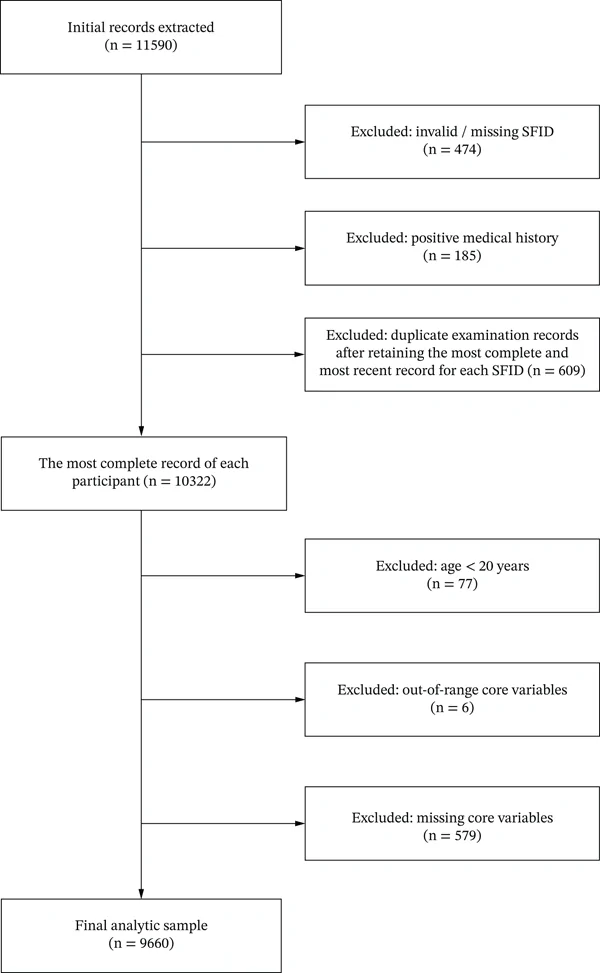
Overview of participant selection and analytic sample. Note: The study window was August 2024 to March 2026. Of the 11,590 source records, 474 with invalid or missing subject identifiers were excluded, followed by 185 linked to a positive medical history. After removing 609 nonretained duplicate examination records, one record was retained for each of the 10,322 participants. We then excluded 77 participants because their age was < 20 years, six because core variables were outside the prespecified plausible ranges, and 579 because core variables were missing, leaving a final analytic sample of 9660 participants.

Participants with MetS were older, more often male, and had higher weight, waist and hip circumferences, systolic and diastolic blood pressures, fasting plasma glucose, triglycerides, low‐density lipoprotein cholesterol, blood urea nitrogen, creatinine, and uric acid, together with lower HDL‐C. All 13 indices were higher in the MetS group (Table 1).

**Table 1:** Baseline characteristics according to IDF‐defined MetS status.

Variable	Overall (*n* = 9660)	No‐MetS (*n* = 8025)	MetS (*n* = 1635)	*p*
Age, years	43.75 (11.47)	42.76 (11.24)	48.60 (11.34)	< 0.001
Sex				< 0.001
Female	4962 (51.4)	4395 (54.8)	567 (34.7)	
Male	4698 (48.6)	3630 (45.2)	1068 (65.3)	
Weight, kg	65.59 (12.88)	63.41 (11.80)	76.31 (12.60)	< 0.001
Height, cm	164.95 (8.60)	164.70 (8.53)	166.20 (8.84)	< 0.001
Waist circumference, cm	83.89 (10.91)	81.60 (9.96)	95.12 (8.03)	< 0.001
Hip circumference, cm	96.42 (6.40)	95.41 (5.98)	101.39 (6.07)	< 0.001
SBP, mmHg	121.14 (16.16)	118.24 (14.58)	135.39 (15.97)	< 0.001
DBP, mmHg	74.33 (11.23)	72.41 (10.19)	83.78 (11.30)	< 0.001
FPG, mmol/L	4.76 [4.45, 5.13]	4.71 [4.42, 5.01]	5.28 [4.77, 5.95]	< 0.001
TG, mmol/L	1.14 [0.81, 1.72]	1.04 [0.77, 1.44]	2.12 [1.71, 2.98]	< 0.001
HDL‐C, mmol/L	1.39 [1.19, 1.62]	1.44 [1.25, 1.67]	1.16 [1.01, 1.31]	< 0.001
TC, mmol/L	5.26 [4.62, 5.95]	5.24 [4.61, 5.90]	5.40 [4.71, 6.15]	< 0.001
LDL‐C, mmol/L	3.25 [2.77, 3.76]	3.21 [2.73, 3.72]	3.45 [2.97, 3.99]	< 0.001
TBIL, *μ*mol/L	13.30 [10.60, 16.83]	13.30 [10.60, 16.80]	13.30 [10.50, 16.90]	0.773
BUN, mmol/L	5.03 [4.27, 5.89]	5.00 [4.24, 5.86]	5.17 [4.43, 6.08]	< 0.001
Cr, *μ*mol/L	68.00 [58.00, 81.00]	67.00 [57.00, 81.00]	73.00 [61.00, 85.00]	< 0.001
UA, *μ*mol/L	333.00 [273.00, 402.00]	322.00 [267.00, 388.00]	389.00 [327.00, 453.00]	< 0.001
TyG	8.40 [8.01, 8.83]	8.27 [7.95, 8.63]	9.12 [8.87, 9.48]	< 0.001
TyG‐WC	703.82 [614.52, 799.24]	674.90 [598.23, 754.18]	869.35 [811.22, 933.85]	< 0.001
TyG‐WHtR	4.27 [3.77, 4.81]	4.10 [3.69, 4.54]	5.20 [4.89, 5.58]	< 0.001
CHG	5.09 [4.88, 5.30]	5.03 [4.84, 5.22]	5.40 [5.24, 5.58]	< 0.001
CHG‐WC	426.44 [372.74, 480.03]	409.65 [362.66, 456.10]	514.41 [478.94, 550.15]	< 0.001
TCBI	1325.24 [822.13, 2255.45]	1154.29 [750.78, 1817.62]	3093.81 [2051.97, 4646.91]	< 0.001
CMI	0.42 [0.25, 0.73]	0.36 [0.23, 0.57]	1.07 [0.77, 1.56]	< 0.001
LAP	25.20 [13.60, 45.54]	20.85 [12.00, 34.73]	68.78 [50.14, 98.91]	< 0.001
NHHR	2.77 [2.13, 3.51]	2.60 [2.03, 3.27]	3.69 [3.09, 4.26]	< 0.001
NHHR‐WC	231.64 [166.27, 310.82]	211.13 [156.60, 280.45]	346.28 [284.93, 413.91]	< 0.001
METS‐IR	33.69 [29.10, 38.99]	32.22 [28.23, 36.48]	43.13 [39.37, 47.60]	< 0.001
VAI	1.29 [0.84, 2.07]	1.13 [0.78, 1.68]	2.85 [2.07, 4.05]	< 0.001
CVAI	78.52 [41.55, 113.74]	67.29 [34.74, 97.81]	132.43 [114.38, 153.81]	< 0.001

*Note:* Age, weight, height, waist circumference, hip circumference, SBP, and DBP are presented as mean (standard deviation); FPG, TG, HDL‐C, TC, LDL‐C, TBIL, BUN, Cr, UA, and the 13 indices are presented as median [interquartile range]; categorical variables are presented as *n* (%). SBP and DBP are reported in mmHg; biochemical variables use mmol/L or *μ*mol/L as indicated. *p* values were calculated using Student′s *t*‐test, Wilcoxon rank‐sum test, chi‐square test, or Fisher′s exact test, as appropriate.

Abbreviations: BUN, blood urea nitrogen; Cr, creatinine; DBP, diastolic blood pressure; FPG, fasting plasma glucose; HDL‐C, high‐density lipoprotein cholesterol; LDL‐C, low‐density lipoprotein cholesterol; MetS, metabolic syndrome; SBP, systolic blood pressure; TBIL, total bilirubin; TC, total cholesterol; TG, triglyceride; UA, uric acid.

### 3.2. Associations Between Indices and MetS

All 13 indices were positively associated with MetS in the unadjusted model and in Models 1 and 2 (all *p* < 0.001; Table 2). In Model 2, CMI showed the strongest standardized association (OR per 1 SD, 19.437; 95% CI, 16.039–23.554), followed by TyG‐WC (16.332; 14.167–18.828) and LAP (15.482; 13.152–18.224). The remaining indices, in descending order, were CVAI, CHG‐WC, TyG‐WHtR, TCBI, VAI, METS‐IR, NHHR‐WC, CHG, TyG, and NHHR. This ranking reflects the standardized association strength rather than discrimination.

**Table 2:** Associations of 13 routinely derived indices with IDF‐defined MetS.

Indicator	Unadjusted OR (95% CI)	Unadjusted *p*	Model 1 OR (95% CI)	Model 1 *p*	Model 2 OR (95% CI)	Model 2 *p*
CMI	22.783 (19.201–27.035)	< 0.001	24.356 (20.195–29.374)	< 0.001	19.437 (16.039–23.554)	< 0.001
TyG‐WC	9.153 (8.235–10.174)	< 0.001	15.666 (13.689–17.928)	< 0.001	16.332 (14.167–18.828)	< 0.001
LAP	15.786 (13.702–18.186)	< 0.001	16.072 (13.813–18.701)	< 0.001	15.482 (13.152–18.224)	< 0.001
CVAI	7.805 (7.055–8.634)	< 0.001	14.729 (12.867–16.861)	< 0.001	13.768 (11.976–15.828)	< 0.001
CHG‐WC	7.581 (6.873–8.362)	< 0.001	11.829 (10.465–13.371)	< 0.001	13.062 (11.435–14.920)	< 0.001
TyG‐WHtR	11.160 (9.943–12.526)	< 0.001	11.479 (10.180–12.944)	< 0.001	12.006 (10.569–13.638)	< 0.001
TCBI	13.262 (11.053–15.913)	< 0.001	12.467 (10.244–15.173)	< 0.001	11.971 (9.545–15.014)	< 0.001
VAI	15.006 (12.898–17.458)	< 0.001	13.948 (11.948–16.282)	< 0.001	11.615 (9.905–13.619)	< 0.001
METS‐IR	7.655 (6.953–8.427)	< 0.001	9.944 (8.889–11.125)	< 0.001	9.470 (8.441–10.624)	< 0.001
NHHR‐WC	4.331 (4.016–4.669)	< 0.001	5.107 (4.672–5.583)	< 0.001	9.280 (8.203–10.498)	< 0.001
CHG	4.917 (4.519–5.349)	< 0.001	4.644 (4.249–5.076)	< 0.001	6.961 (6.218–7.792)	< 0.001
TyG	5.763 (5.287–6.283)	< 0.001	5.723 (5.224–6.269)	< 0.001	5.932 (5.376–6.546)	< 0.001
NHHR	3.185 (2.976–3.409)	< 0.001	3.158 (2.933–3.401)	< 0.001	4.891 (4.415–5.419)	< 0.001

*Note:* *N* = 9660 for all models. ORs and 95% CIs are reported per 1‐standard‐deviation increment for each index. The unadjusted model included only the index. Model 1 was adjusted for age and sex, and Model 2 was additionally adjusted for LDL‐C, TBIL, BUN, Cr, and UA. Estimates are reported to three decimal places.

Abbreviations: CI, confidence interval; IDF, International Diabetes Federation; OR, odds ratio.

### 3.3. Discrimination and Subgroup Analyses

The association and discrimination rankings differed. In the unadjusted analysis, TyG‐WHtR had the highest AUC (0.924; 95% CI, 0.919–0.930), followed by LAP (0.915), and TyG‐WC (0.911) (Figure S1 and Table S2).

After adjusting for age and sex in Model 1, TyG‐WC had the highest AUC (0.933; 95% CI, 0.927–0.937), followed by TyG‐WHtR (0.926) and METS‐IR (0.922) (Figure S2 and Table S3).

In Model 2, TyG‐WC had the highest AUC (0.934; 95% CI, 0.929–0.939), with a reference‐profile equivalent index value of 725.825 at the Youden‐selected probability threshold, a sensitivity of 0.930, and a specificity of 0.810. The remaining indices in descending AUC order were TyG‐WHtR (0.928), METS‐IR (0.923), CHG‐WC (0.921), LAP (0.920), CVAI (0.915), CMI (0.903), TyG (0.902), VAI (0.896), NHHR‐WC (0.890), CHG (0.885), TCBI (0.863), and NHHR (0.852) (Figure 2 and Table 3). Notably, composite forms incorporating central adiposity information generally showed higher discrimination than their simpler counterparts: TyG‐WC and TyG‐WHtR exceeded TyG, CHG‐WC exceeded CHG, and NHHR‐WC exceeded NHHR.

**Figure 2:**
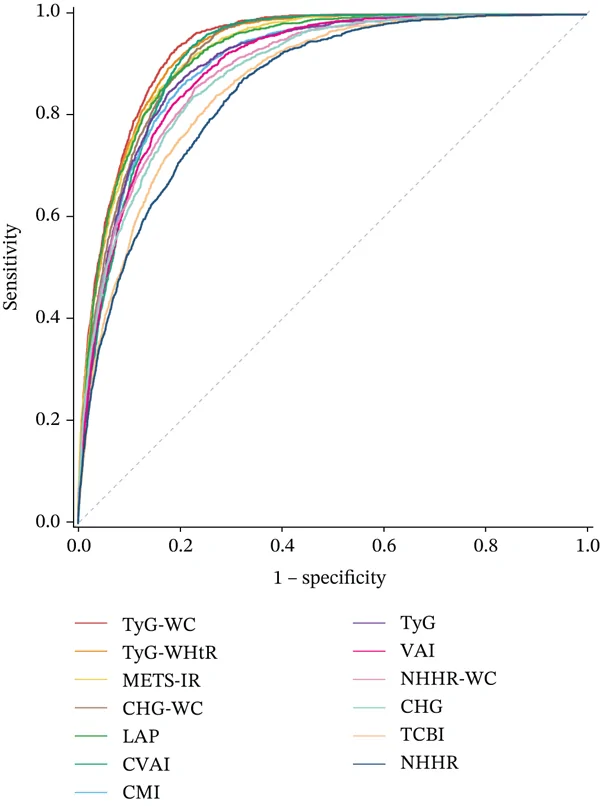
Model 2 ROC curves for 13 routinely derived metabolic indices in the overall analytic sample. Note: Model 2 was adjusted for age, sex, LDL‐C, TBIL, BUN, Cr, and UA. The adjusted ROC curves were based on model‐predicted probabilities. Abbreviations: ROC, receiver operating characteristic curve; IDF, International Diabetes Federation; AUC, area under the curve.

**Table 3:** Model 2 ROC performance and reference‐profile equivalent index values of 13 indices for identifying IDF‐defined MetS.

Indicator	AUC	95% CI	Reference‐profile equivalent index value	Sensitivity	Specificity
TyG‐WC	0.934	0.929–0.939	725.825	0.930	0.810
TyG‐WHtR	0.928	0.922–0.933	4.617	0.893	0.823
METS‐IR	0.923	0.917–0.929	36.576	0.900	0.794
CHG‐WC	0.921	0.915–0.926	429.124	0.922	0.777
LAP	0.920	0.914–0.926	44.719	0.876	0.814
CVAI	0.915	0.909–0.920	79.363	0.938	0.775
CMI	0.903	0.895–0.910	0.694	0.861	0.796
TyG	0.902	0.895–0.909	8.724	0.847	0.822
VAI	0.896	0.889–0.903	2.501	0.837	0.800
NHHR‐WC	0.890	0.883–0.898	249.788	0.856	0.770
CHG	0.885	0.877–0.893	5.154	0.839	0.774
TCBI	0.863	0.855–0.871	2498.535	0.842	0.727
NHHR	0.852	0.843–0.861	2.874	0.875	0.674

*Note:* *N* = 9660. Model 2 was adjusted for age, sex, LDL‐C, TBIL, BUN, Cr, and UA. The adjusted ROC analyses were based on model‐predicted probabilities. Reference‐profile equivalent index values correspond to the Youden‐selected probability thresholds with continuous covariates fixed at their sample medians and categorical covariates at their modal levels. These values are not direct univariable ROC cutoffs and are not clinically validated diagnostic thresholds. AUCs, 95% CIs, reference‐profile equivalent index values, sensitivity, and specificity are reported to three decimal places.

Abbreviations: AUC, area under the curve; CI, confidence interval; IDF, International Diabetes Federation; ROC, receiver operating characteristic curve.

TyG‐WC had the highest AUC in 9 of 11 prespecified age, sex, and age‐by‐sex strata. Among women overall, TyG‐WC ranked first (AUC, 0.949), with METS‐IR showing comparable discrimination (AUC, 0.949), while LAP ranked first among women aged 20–44 years (0.976) and CHG among women aged ≥ 65 years (0.888). Complete rankings and the reference‐profile equivalent index values for the three leading indices in each stratum are provided in Tables S4 and S5 and Figure S3.

### 3.4. Restricted Cubic Splines, Collinearity Diagnostics, and Robustness Analyses

Restricted cubic spline analyses showed significant overall and nonlinear associations between all 13 indices and IDF‐defined MetS (all *p* for overall and nonlinearity < 0.001; Figure 3 and Table S6). Overall, the odds of MetS increased with higher index levels, although the magnitude and steepness of the associations and their patterns at higher values differed across the indices. Model diagnostics did not indicate substantial multicollinearity, with a maximum VIF of 2.768 across the 13 exposure‐specific models (Table S7).

**Figure 3:**
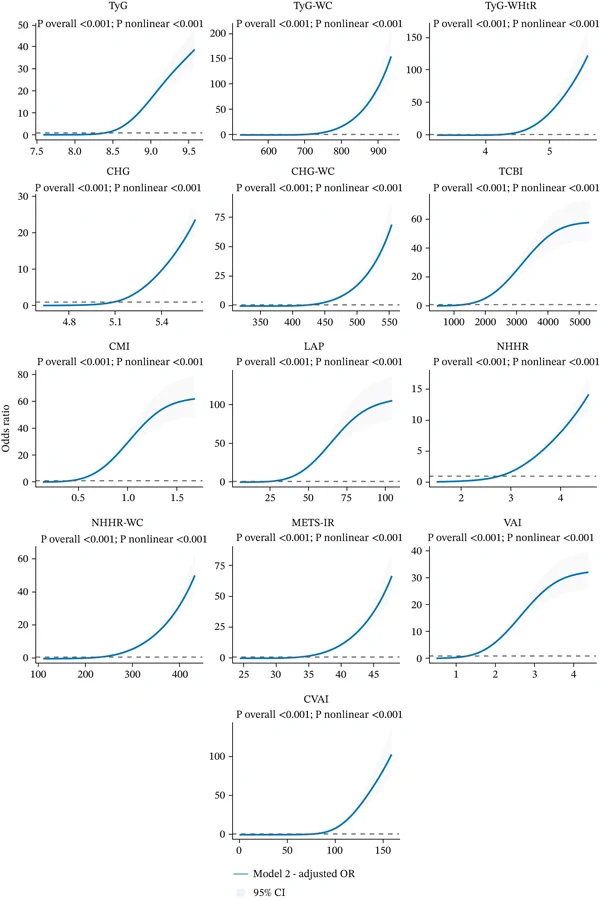
Model 2 restricted cubic spline associations of 13 routinely derived metabolic indices with IDF‐defined MetS. Note: Each exposure was modeled using four knots. Model 2 was adjusted for age, sex, LDL‐C, TBIL, BUN, Cr, and UA. ORs were referenced to the median of each index; the dashed horizontal line indicates an OR of 1. Solid lines represent Model 2–adjusted ORs, and shaded areas indicate 95% CIs. Overall and nonlinear *p* values are reported within each panel. Abbreviations: RCS, restricted cubic spline; IDF, International Diabetes Federation.

Winsorized sensitivity analyses showed that the standardized association estimates were affected differently across indices and scaling approaches, whereas discrimination was generally more stable. All associations with MetS remained positive under both standardization approaches (all *p* < 0.001). Under self‐SD scaling, CMI, TCBI, VAI, and LAP showed substantial decreases in standardized ORs, with changes in excess OR (OR−1) of −79.3%, −72.2%, −70.8%, and −62.2%, respectively. Under fixed main‐SD scaling, the corresponding changes were +63.9%, +396.0%, +49.3%, and +58.8%, respectively (Table S8 and Figure S4). These four indices also showed pronounced positive skewness before winsorization (15.374–30.571), which was markedly reduced after winsorization (2.813–3.552), together with substantial reductions in SD. These findings indicate that their per‐SD OR estimates were highly sensitive to changes in the index dispersion and standardization scale and should not be interpreted as showing uniform weakening or strengthening of the underlying associations. In contrast, TyG‐WC showed comparatively limited distributional and scale‐related variations and retained strong standardized associations, with Model 2 ORs of 16.332 in the main analysis, 17.181 under self‐SD scaling, and 18.638 under fixed main‐SD scaling. Its discrimination was similarly stable, with the AUC changing only from 0.934 to 0.935 after winsorization. The overall AUC rankings remained largely stable, with TyG‐WC retaining the highest discrimination (Table S9). TCBI provided a notable contrast between association‐scale sensitivity and discrimination; despite marked variation in its standardized OR across the two scaling approaches, its AUC increased from 0.863 to 0.881, the largest numerical AUC change among the 13 indices. Further component‐specific analyses indicated that this increase was driven primarily by handling of the upper tail of TG: winsorizing TG alone increased the TCBI AUC from 0.863 to 0.880 (*Δ*AUC = 0.017), nearly accounting for the change observed when TG, TC, and body weight were all winsorized (AUC = 0.881; *Δ*AUC = 0.018). In comparison, winsorizing TC alone yielded an AUC of 0.872 (*Δ*AUC = 0.009), whereas winsorizing body weight alone produced essentially no change (AUC = 0.863; *Δ*AUC = 0.000) (Table S10).

## 4. Discussion

### 4.1. Principal Findings

This head‐to‐head comparison showed that the association strength and discrimination did not identify the same leading index. CMI had the strongest standardized association in the main analysis, whereas TyG‐WC had the second strongest standardized association, the highest overall AUC, and the highest AUC in 9 of 11 prespecified strata. The two parallel winsorized standardization analyses further showed that the per‐SD OR estimates for several indices, particularly CMI, LAP, VAI, and TCBI, were strongly influenced by the postwinsorization dispersion and standardization scale. In contrast, TyG‐WC showed comparatively limited changes in distribution and standardized ORs, while retaining stable and leading discrimination. These findings support the interpretation of association strength, discrimination, subgroup patterns, and distributional and scaling robustness separately rather than relying on a single performance metric.

### 4.2. Comparative Interpretation of Different Metabolic Constructs

The 13 indices overlap in their component structure and metabolic emphasis; however, they also capture distinct aspects of metabolic dysfunction. The RCS analyses further showed significant nonlinear associations with MetS across all 13 indices. Taken together, the different metabolic constructs showed distinct patterns of association strength, discrimination, and distributional robustness, indicating that their performance could not be adequately characterized by any single statistical measure. Therefore, a comparison of these three broad groups provides a useful framework for understanding these differences and their potential sources.

Indices primarily reflecting glycemic lipid metabolism and insulin resistance‐related information showed the strongest overall discrimination. Previous studies have linked TyG and its adiposity‐modified forms to MetS and other cardiometabolic phenotypes [5, 6], whereas CHG and METS‐IR capture complementary metabolic information through cholesterol‐glucose combinations and noninsulin‐based surrogates of insulin resistance, respectively [11, 14–16]. In the present study, this group contained the four indices with the highest overall AUCs: TyG‐WC, TyG‐WHtR, METS‐IR, and CHG‐WC. Within‐group comparisons further showed that TyG‐WC and TyG‐WHtR had higher AUCs than TyG (0.934 and 0.928 vs. 0.902, respectively), and CHG‐WC similarly outperformed CHG (0.921 vs. 0.885, respectively). These findings indicate that incorporating waist‐related adiposity information into glycemic lipid constructs may further improve the discrimination of MetS. Standardized association estimates for most indices in this group also showed comparatively limited variation between the two winsorized standardization approaches. Nevertheless, because central obesity is itself a key component of the IDF definition, the higher discrimination observed for waist‐related indices may partly reflect the overlap between index components and the outcome definition, and should therefore be interpreted in the context of potential incorporation bias.

Indices characterized primarily by central fat distribution and lipid accumulation showed stronger standardized associations. CMI, LAP, VAI, and CVAI represent this metabolic domain through different combinations of TG/HDL‐C, body size measures, lipid accumulation, and visceral adiposity‐related characteristics [7–13]. CMI showed the strongest standardized association among all 13 indices (OR, 19.437); LAP and CVAI also showed strong associations. Their discriminatory performances remained generally good, although they did not reach those of the leading indices in the first group. Notably, CMI, LAP, and VAI showed pronounced positive skewness before winsorization, with marked reductions in both skewness and SD after winsorization. This pattern may partly reflect their multiplicative and ratio‐based formulations, particularly their dependence on TG and, for CMI and VAI, their inverse dependence on HDL‐C, which can amplify upper‐tail values. Consistent with these distributional changes, their standardized ORs varied substantially between the self‐SD and fixed main‐SD approaches. These divergent estimates should not be interpreted as evidence that winsorization uniformly weakened or strengthened the underlying associations. Overall, these indices combined strong associations with good discrimination; however, their standardized association estimates were less uniform across distributional handling and scaling, further illustrating that a larger standardized OR does not necessarily translate into higher or more stable discrimination.

Overall, indices primarily reflecting lipid burden and lipoprotein balance showed comparatively weaker associations and discrimination. TCBI and NHHR emphasize composite lipid‐weight burden and atherogenic lipoprotein balance, respectively, and have been evaluated in previous cardiometabolic studies [17–20]. Within this group, the waist‐modified NHHR‐WC showed higher discrimination than NHHR (AUC, 0.890 vs. 0.852). This pattern was directionally consistent with the higher discrimination observed for other waist‐modified indices, suggesting that central adiposity information may contribute additional discriminatory information beyond lipoprotein balance alone, although part of this advantage may reflect an overlap with the waist circumference component of the IDF MetS definition. TCBI provided a particularly clear illustration of the distinction between scale‐sensitive standardized association estimates and discriminatory performance. The per‐SD OR varied markedly between self‐SD and fixed main‐SD scaling, indicating that the magnitude of the standardized association was sensitive to the scaling approach. In contrast, AUC was unaffected by the choice of SD scaling. The increase in TCBI AUC from 0.863 in the main analysis to 0.881 after winsorization (*Δ*AUC = 0.018), which was the largest numerical change among the 13 indices, therefore reflected the effect of winsorization rather than the standardization scale. Its multiplicative formulation, in which TG, TC, and body weight are multiplied, may partially amplify extreme upper‐tail values. Component‐specific analyses further showed that winsorizing TG alone produced an AUC change of 0.017, nearly accounting for the total change observed when all TCBI components were winsorized (*Δ*AUC = 0.018), indicating that the upper tail of TG was the principal driver of the discrimination change. These findings suggest that product‐based indices may be particularly sensitive to the upper tail distribution of individual components.

Taken together, under the study‐specific descriptive grouping used in this study, the three metabolic constructs showed a relatively clear, although not absolute, overall pattern. Glycemic lipid/insulin resistance‐related indices were generally more prominent in terms of discrimination and showed comparatively favorable distributional and scaling robustness, particularly when combined with central adiposity information. Central adiposity/lipid accumulation‐related indices tended to show stronger standardized associations but somewhat lower discrimination, with several indices also showing greater sensitivity of per‐SD association estimates to distributional handling and scaling. Lipid‐burden/lipoprotein–balance‐related indices showed comparatively weaker associations and discrimination overall.

These group‐level patterns should be interpreted as within‐sample findings under a study‐specific descriptive framework, rather than as an established taxonomy. As several indices share components and may span more than one metabolic domain, alternative grouping schemes can yield different group‐level patterns. Therefore, caution is warranted when extrapolating these patterns to other populations or to alternative definitions of MetS.

This cross‐group pattern also helps place the relative performance of CMI and TyG‐WC in context. CMI had the strongest standardized association in the main analysis (OR 19.437), but its distribution changed markedly after winsorization, and its per‐SD OR varied substantially between self‐SD and fixed main‐SD scaling. In contrast, TyG‐WC had the second‐strongest standardized association in the main analysis (OR 16.332), showed comparatively limited distributional changes after winsorization, and retained strong standardized associations under both winsorized scaling approaches (ORs 17.181 and 18.638). TyG‐WC had the highest overall AUC in both the main and winsorized analyses (0.934 and 0.935, respectively). This contrast may partly reflect the formula structure, as the logarithmic transformation within TyG compresses extreme TG and fasting glucose values before combining with waist circumference. Thus, TyG‐WC did not dominate every individual statistical measure but combined a consistently strong standardized association with the highest and highly stable overall discrimination and comparatively favorable distributional and scaling stability. This combination, rather than the superiority of any single estimate, supports a more balanced overall profile. Therefore, these comparisons characterized the performance within the existing IDF‐defined MetS framework.

### 4.3. Subgroup Interpretation

TyG‐WC had the highest AUC overall and in 9 of 11 prespecified strata. Among participants aged 20–44 years, TyG‐WC showed the highest discrimination overall and in men, whereas LAP ranked first in women. Among those aged 45–64 years, TyG‐WC showed the highest AUC overall, and in both men and women. These patterns support TyG‐WC as a principal candidate for further evaluation in stratified screening across most nonelderly health examination groups, whereas LAP may warrant particular attention among younger women. Among women overall, METS‐IR showed discrimination comparable with that of TyG‐WC (both AUCs, 0.949). Among participants aged ≥ 65 years, TyG‐WC ranked first overall and in men, whereas CHG ranked first in women. However, the ≥ 65‐year subgroup, particularly after sex stratification, was relatively small; findings in these older‐adult strata should therefore be regarded as exploratory and hypothesis‐generating. External validation is necessary before any age‐ or sex‐specific index selection or reference‐profile equivalent index value can be adopted in practice.

### 4.4. Health‐Examination Information Systems and Clinical Implications

Because all 13 indices can be calculated from routinely collected anthropometric, glucose, and lipid data, they can be automatically derived within health examination information systems to facilitate the routine review of metabolic profiles. Given its balanced performance across association strength, discrimination, and robustness, TyG‐WC may merit further evaluation in this setting. Any future implementation would require standardized calculations, measurement quality control, local calibration, and external validation.

### 4.5. Strengths and Limitations

This study has several strengths. A relatively large routine health examination sample, single IDF outcome definition, and common covariate framework enabled a direct head‐to‐head comparison of 13 indices with different metabolic constructs. Evaluating standardized associations, ROC discrimination, subgroup performance, nonlinearity, and distributional robustness provided complementary views of performance rather than reducing comparison to a single statistic. Compared with studies that focused on a single index or construct, this design better delineates the relative strengths and boundaries of routinely derived indices.

Several limitations of this study should be considered. First, its cross‐sectional design precluded the assessment of temporal sequences and did not support causal inferences. Accordingly, the present analysis reflects the cross‐sectional recognition of the existing IDF‐defined MetS phenotype rather than the prospective prediction of incident MetS. Second, the study was conducted in a single‐center hospital‐based health examination population, and examinees with self‐reported histories of relevant diseases, major surgery, or treatment were excluded, resulting in a potentially healthier analytic sample. The observed MetS prevalence of 16.93% reflects this specific case mix and risk distribution, both of which may influence AUC estimates and reference‐profile equivalent index values. Therefore, the findings should not be directly generalized to the general population, other regions, or populations with different risk and disease spectra. Reference‐profile equivalent index values require validation in independent multicenter populations and should not be interpreted as clinically validated diagnostic thresholds. Third, MetS status was determined for all participants using the same prespecified IDF algorithm, making classic partial verification bias arising from the selective application of a reference standard unlikely to be a major concern. However, several evaluated indices share components with the IDF definition, including waist circumference, triglycerides, HDL‐C, and fasting glucose, creating the potential for incorporation bias. Therefore, the high AUCs observed in this study should be interpreted primarily as cross‐sectional discrimination of an existing IDF‐defined diagnostic phenotype rather than as evidence of independent etiologic or prognostic prediction. In addition, the three broad groups were study‐specific descriptive categories rather than an established classification system, and some indices span overlapping metabolic domains; therefore, the grouping framework and the observed group‐level performance patterns require further evaluation in other populations and under alternative MetS definitions. Fourth, detailed information on medication use, diet, physical activity, smoking, alcohol consumption, and socioeconomic factors was not comprehensively available, leaving the possibility of residual confounding. Finally, the ≥ 65‐year subgroup, particularly after sex stratification, was relatively small; therefore, findings in these older‐adult strata should be regarded as exploratory and hypothesis‐generating.

## 5. Conclusions

Among 9660 Chinese adults undergoing routine health examinations, all 13 routinely derived indices were positively associated with IDF‐defined MetS, with significant nonlinear patterns across index levels, but differed in the strength of association, discriminatory performance, and distributional robustness. Under the study‐specific grouping used in this study, glycemic‐lipid/insulin–resistance‐related indices showed more favorable discrimination and robustness; central adiposity/lipid accumulation‐related indices tended to exhibit stronger standardized associations, although some showed greater sensitivity of standardized association estimates to distributional handling and scaling; lipid‐burden/lipoprotein–balance‐related indices showed comparatively weaker associations and discrimination. Among the individual indices, TyG‐WC demonstrated the most balanced overall profile in terms of association strength, discrimination, and distributional robustness. These findings support further evaluation of TyG‐WC in routine health examination settings. The observed group‐level patterns and balanced overall profile of TyG‐WC require further validation in independent multicenter populations.

## Author Contributions

**Qiuman Lin:** conceptualization, methodology, writing – original draft. **Fen Zhou:** data curation, formal analysis, visualization, writing – review and editing. **Yongjian Zhang:** conceptualization, methodology, writing – review and editing. **Qianqian Zhong:** investigation, validation, writing – review and editing. **Jianxin Liu:** supervision, methodology, writing – review and editing. **Qiuman Lin** and **Fen Zhou** have contributed to the work equally and should be regarded as co‐first authors.

## Funding

No funding was received for this manuscript.

## Disclosure

All authors have read and approved the final version of the manuscript. Jianxin Liu, the corresponding author and manuscript guarantor, had full access to all of the data in this study and takes complete responsibility for the integrity of the data and the accuracy of the data analysis. The final analyses and visualizations were performed and verified by the authors. The authors retain full responsibility for the scientific content, analyses, interpretations, and conclusions of the manuscript.

## Ethics Statement

The Research Ethics Committee of Peking University Shenzhen Hospital approved this study (Peking University Shenzhen Hospital Ethics Review [Research] [2026] No. 102). The study was conducted in accordance with the Declaration of Helsinki and the approved institutional protocol.

## Consent

The Research Ethics Committee of Peking University Shenzhen Hospital waived the requirement for individual informed consent because this retrospective analysis used existing routine health‐examination records and involved no additional intervention.

## Conflicts of Interest

The authors declare no conflicts of interest.

## General Statement

*Related Manuscript Disclosure.* A related manuscript derived from the same de‐identified health‐examination database is under separate consideration. That study evaluates conventional obesity and body‐shape indices together with a broader family of TyG‐related anthropometric composite indices, whereas the present study compares 13 routinely derived metabolic indices representing different metabolic constructs. TyG‐WC and TyG‐WHtR are included in both studies as representative waist‐based TyG composite indices within distinct comparative frameworks. Although the two studies share the source population and IDF‐defined metabolic syndrome outcome, their exposure frameworks, primary comparisons, analyses, tables, figures, interpretations, and conclusions are distinct. No primary analysis, table, or figure is duplicated between the manuscripts.

## Supporting information


**Supporting Information** Additional supporting information can be found online in the Supporting Information section. Table S1: Formulae and input‐unit conventions for the 13 routinely derived metabolic indices. Table S2: Unadjusted ROC performance and sample‐specific operating points of 13 indices for identifying IDF‐defined MetS. Table S3: Model 1 ROC performance and sample‐specific probability operating points of 13 indices for identifying IDF‐defined MetS. Table S4: Ranking of AUCs for all 13 indices across 11 prespecified age, sex, and combined age‐sex strata under Model 2. Table S5: Top three indices and reference‐profile equivalent index values within each prespecified stratum under Model 2. Table S6: Overall and nonlinear associations between 13 indices and IDF‐defined MetS based on restricted cubic splines. Table S7: Exposure‐specific variance inflation factor diagnostics for Model 2. Table S8: Main and winsorized Model 2 logistic associations under two parallel standardization approaches. Table S9: Main versus winsorized overall Model 2 ROC performance. Table S10: Distributional effects of component‐specific winsorization on TCBI and its Model 2 ROC discrimination. Figure S1: Unadjusted ROC curves for 13 indices. Figure S2: Model 1 ROC curves for 13 indices. Figure S3: Model 2 ROC curves across 11 prespecified age, sex, and age‐by‐sex strata. Figure S4: Model 2 logistic associations in the main analysis and the two winsorized standardization approaches.

## Data Availability

The participant‐level data analyzed in this study are not publicly available because of privacy and institutional restrictions. Deidentified data may be made available from the corresponding author upon reasonable request and subject to institutional approval.

## References

[1] Noubiap J. J. , Nansseu J. R. , Lontchi-Yimagou E. , Nkeck J. R. , Nyaga U. F. , Ngouo A. T. , Tounouga D. N. , Tianyi F. L. , Foka A. J. , Ndoadoumgue A. L. , and Bigna J. J. , Geographic Distribution of Metabolic Syndrome and Its Components in the General Adult Population: A Meta-Analysis of Global Data from 28 Million Individuals, Diabetes Research and Clinical Practice. (2022) 188, 109924, 10.1016/j.diabres.2022.109924, 35584716. 35584716

[2] Alberti K. G. , Zimmet P. , and Shaw J. , The Metabolic Syndrome--A New Worldwide Definition, Lancet. (2005) 366, no. 9491, 1059–1062, 10.1016/s0140-6736(05)67402-8. 16182882

[3] Alberti K. G. , Zimmet P. , and Shaw J. , Metabolic Syndrome--A New World-Wide Definition. A Consensus Statement From the International Diabetes Federation, Diabetic Medicine. (2006) 23, no. 5, 469–480, 10.1111/j.1464-5491.2006.01858.x. 16681555

[4] Guerrero-Romero F. , Simental-Mendía L. E. , González-Ortiz M. , Martínez-Abundis E. , Ramos-Zavala M. G. , Hernandez-Gonzalez S. O. , Jacques-Camarena O. , and Rodríguez-Moran M. , The Product of Triglycerides and Glucose, a Simple Measure of Insulin Sensitivity. Comparison With the Euglycemic-Hyperinsulinemic Clamp, Journal of Clinical Endocrinology and Metabolism. (2010) 95, no. 7, 3347–3351, 10.1210/jc.2010-0288, 20484475. 20484475

[5] Dang K. , Wang X. , Hu J. , Zhang Y. , Cheng L. , Qi X. , Liu L. , Ming Z. , Tao X. , and Li Y. , The Association Between Triglyceride-Glucose Index and Its Combination With Obesity Indicators and Cardiovascular Disease: NHANES 2003-2018, Cardiovascular Diabetology. (2024) 23, no. 1, 10.1186/s12933-023-02115-9, 38184598. PMC10771672 38184598

[6] Glasgow A. C. , Heckel A. R. , Cho W. , and Kim J. Y. , Can Adiposity Measures Enhance the Predictive Power of the Triglyceride-Glucose Index for Metabolic Syndrome in Adults in the United States?, Obesity Research & Clinical Practice. (2024) 18, no. 5, 343–349, 10.1016/j.orcp.2024.11.001, 39500652. 39500652

[7] Tamini S. , Bondesan A. , Caroli D. , and Sartorio A. , The Lipid Accumulation Product Index (LAP) and the Cardiometabolic Index (CMI) Are Useful for Predicting the Presence and Severity of Metabolic Syndrome in Adult Patients With Obesity, Journal of Clinical Medicine. (2024) 13, no. 10, 10.3390/jcm13102843, 38792386. PMC11122168 38792386

[8] Kahn H. S. , The "Lipid Accumulation Product" Performs Better Than the Body Mass Index for Recognizing Cardiovascular Risk: A Population-Based Comparison, BMC Cardiovascular Disorders. (2005) 5, no. 1, 10.1186/1471-2261-5-26, 16150143. PMC1236917 16150143

[9] Amato M. C. , Giordano C. , Galia M. , Criscimanna A. , Vitabile S. , Midiri M. , Galluzzo A. , and for the AlkaMeSy Study Group , Visceral Adiposity Index: A Reliable Indicator of Visceral Fat Function Associated With Cardiometabolic Risk, Diabetes Care. (2010) 33, no. 4, 920–922, 10.2337/dc09-1825, 20067971. PMC2845052 20067971

[10] Duan S. , Yang D. , Xia H. , Ren Z. , Chen J. , and Yao S. , Cardiometabolic Index: A New Predictor for Metabolic Associated Fatty Liver Disease in Chinese Adults, Frontiers in Endocrinology. (2022) 13, 1004855, 10.3389/fendo.2022.1004855, 36187093. PMC9523727 36187093

[11] Bello-Chavolla O. Y. , Almeda-Valdes P. , Gomez-Velasco D. , Viveros-Ruiz T. , Cruz-Bautista I. , Romo-Romo A. , Sánchez-Lázaro D. , Meza-Oviedo D. , Vargas-Vázquez A. , Campos O. A. , Sevilla-González M. D. R. , Martagón A. J. , Hernández L. M. , Mehta R. , Caballeros-Barragán C. R. , and Aguilar-Salinas C. A. , METS-IR, a Novel Score to Evaluate Insulin Sensitivity, Is Predictive of Visceral Adiposity and Incident Type 2 Diabetes, European Journal of Endocrinology. (2018) 178, no. 5, 533–544, 10.1530/eje-17-0883, 29535168. 29535168

[12] Xia M. F. , Chen Y. , Lin H. D. , Ma H. , Li X. M. , Aleteng Q. , Li Q. , Wang D. , Hu Y. , Pan B. S. , Li X. J. , Li X. Y. , and Gao X. , A Indicator of Visceral Adipose Dysfunction to Evaluate Metabolic Health in Adult Chinese, Scientific Reports. (2016) 6, no. 1, 38214, 10.1038/srep38214, 27905531. PMC5131270 27905531

[13] Gui J. , Li Y. , Liu H. , Guo L. L. , Li J. , Lei Y. , Li X. , Sun L. , Yang L. , Yuan T. , Wang C. , Zhang D. , Wei H. , Li J. , Liu M. , Hua Y. , and Zhang L. , Obesity- and Lipid-Related Indices as a Predictor of Obesity Metabolic Syndrome in a National Cohort Study, Frontiers in Public Health. (2023) 11, 1073824, 10.3389/fpubh.2023.1073824, 36875382. PMC9980350 36875382

[14] Mansoori A. , Nosrati M. , Dorchin M. , Mohammadyari F. , Derakhshan-Nezhad E. , Ferns G. , Esmaily H. , and Ghayour-Mobarhan M. , A Novel Index for Diagnosis of Type 2 Diabetes Mellitus: Cholesterol, High Density Lipoprotein, and Glucose (CHG) Index, Journal of Diabetes Investigation. (2025) 16, no. 2, 309–314, 10.1111/jdi.14343, 39569998. PMC11786182 39569998

[15] Tang J. , Wei S. , Li Y. , and Zhou Y. , Assessment of Cholesterol-HDL-Glucose Index in Anticipating Risk of Cardiometabolic Diseases: A Comparative Study With Triglyceride-Glucose Index, Scientific Reports. (2025) 15, no. 1, 45356, 10.1038/s41598-025-30024-2, 41291178. PMC12748693 41291178

[16] Wei S. , Yu S. , Qian C. , Xu Z. , Jia Y. , and Chen B. , Exploring the U-Shaped Nonlinear Relationship of CHG Index With Metabolic Syndrome and Mortality Risks in Metabolic Syndrome Patients, Lipids in Health and Disease. (2025) 24, no. 1, 10.1186/s12944-025-02714-3, 41023729. PMC12482814 41023729

[17] Rezaee M. , Kamrani F. , Imannezhad M. , Shahri H. H. , Saihood W. K. , Rezvani A. , Far P. M. , Mahaki H. , Esmaily H. , Moohebati M. , Shariati M. , Ghayour-Mobarhan M. , and Darroudi S. , Beyond Traditional Metrics: Evaluating the Triglyceride-Total Cholesterol-Body Weight Index (TCBI) in Cardiovascular Risk Assessment, BMC Cardiovascular Disorders. (2025) 25, no. 1, 10.1186/s12872-025-04500-6, 39849378. PMC11756170 39849378

[18] Fan L. , Su Y. , Chen Y. , Xu L. , Huang H. , Lu C. , Peng J. , Sun Y. , and Jia M. , Triglyceride-Cholesterol-Body Weight Index Associated With the Risk of Metabolic Dysfunction-Associated Steatotic Liver Disease: A Population-Based Cross-Sectional Study, Frontiers in Nutrition. (2025) 12, 1698297, 10.3389/fnut.2025.1698297, 41245409. PMC12611659 41245409

[19] Liang J. , Xie Y. , Li P. , Li H. , Li P. , Huang Z. , Liu G. , Zhong Y. , Li B. , Zhang J. , and Wen J. , The Non-High-Density Lipoprotein Cholesterol to High-Density Lipoprotein Cholesterol Ratio and Its Combination With Obesity Indicators as a Predictor of All Cause and Cardiovascular Mortality in Non-Diabetic Individuals, BMC Public Health. (2025) 25, no. 1, 10.1186/s12889-025-22789-y, 40269817. PMC12016409 40269817

[20] Yu B. , Li M. , Yu Z. , Zheng T. , Feng X. , Gao A. , Zhang H. , and Gao R. , The Non-High-Density Lipoprotein Cholesterol to High-Density Lipoprotein Cholesterol Ratio (NHHR) as a Predictor of All-Cause and Cardiovascular Mortality in US Adults With Diabetes or Prediabetes: NHANES 1999-2018, BMC Medicine. (2024) 22, no. 1, 10.1186/s12916-024-03536-3, 39113030. PMC11304565 39113030

