# Supplementary material for: Routinely Derived Metabolic Indices for IDF‐Defined Metabolic Syndrome in Chinese Adults: A Cross‐Sectional Study
Source: J Diabetes Res. 2026 Oct 16;2026:4648598. doi: 10.1155/jdr/4648598 (PMC13673509; doi:10.1155/jdr/4648598)
Supplement: Supplementary file 1 [file JDR-2026-4648598-s001.docx]

**Supplementary Material**

This supplementary material provides the complete formulae and input-unit conventions for the 13 indices, additional receiver operating characteristic (ROC) analyses, subgroup operating references, restricted cubic spline and collinearity diagnostics, and winsorized sensitivity analyses for 9,660 adults. The winsorized logistic analyses used parallel self-SD and fixed main-SD standardization, while overall winsorized ROC discrimination was evaluated using the recalculated indices.

Supplementary Table 1. Formulae and input-unit conventions for the 13 routinely derived metabolic indices

Supplementary Table 2. Unadjusted ROC performance and sample-specific operating points of 13 indices for identifying IDF-defined MetS.

Supplementary Table 3. Model 1 ROC performance and sample-specific probability operating points of 13 indices for identifying IDF-defined MetS.

Supplementary Table 4. Ranking of AUCs for all 13 indices across 11 prespecified age, sex, and combined age-sex strata under Model 2.

Supplementary Table 5. Top three indices and reference-profile equivalent index values within each prespecified stratum under Model 2.

Supplementary Table 6. Overall and nonlinear associations between 13 indices and IDF-defined MetS based on restricted cubic splines.

Supplementary Table 7. Exposure-specific variance inflation factor diagnostics for Model 2.

Supplementary Table 8. Main and winsorized Model 2 logistic associations under two parallel standardization approaches.

Supplementary Table 9. Main versus winsorized overall Model 2 ROC performance.

Supplementary Table 10. Distributional effects of component-specific winsorization on TCBI and its Model 2 ROC discrimination.

Supplementary Figure 1. Unadjusted ROC curves for 13 indices.

Supplementary Figure 2. Model 1 ROC curves for 13 indices.

Supplementary Figure 3. Model 2 ROC curves across 11 prespecified age, sex, and age-by-sex strata.

Supplementary Figure 4. Model 2 logistic associations in the main analysis and the two winsorized standardization approaches.

Supplementary Table 1. Formulae and input-unit conventions for the 13 routinely derived metabolic indices.

| **Indicator** | **Formula** | **Input units / notes** |
| --- | --- | --- |
| TyG | ln({[TG (mmol/L) × 88.57] × [FPG (mmol/L) × 18]}/2) | TG and FPG entered in mmol/L and converted inside the published formula |
| TyG-WC | TyG × WC (cm) | TyG as defined above; WC in cm |
| TyG-WHtR | TyG × [WC (cm)/height (cm)] | TyG as defined above; WC and height both in cm |
| CHG | ln({[TC (mmol/L) × 38.67] × [FPG (mmol/L) × 18]}/{2 × [HDL-C (mmol/L) × 38.67]}) | TC, FPG, and HDL-C entered in mmol/L and converted inside the published formula |
| CHG-WC | CHG × WC (cm) | CHG as defined above; WC in cm |
| METS-IR | ln{[TG (mmol/L) × 88.57] + 2 × [FPG (mmol/L) × 18]} × BMI / ln[HDL-C (mmol/L) × 38.67] | TG, FPG, and HDL-C entered in mmol/L and converted inside the published formula; BMI in kg/m² |
| CMI | [TG (mmol/L)/HDL-C (mmol/L)] × [WC (cm)/height (cm)] | TG and HDL-C in mmol/L; WC and height in cm |
| LAP | Male: [WC (cm) − 65] × TG (mmol/L); Female: [WC (cm) − 58] × TG (mmol/L) | TG in mmol/L; WC in cm; sex-specific equation. To avoid non-positive LAP values, WC ≤65 cm in men is set to 66.0 cm and WC ≤58 cm in women is set to 59.0 cm before calculation |
| VAI | Male: WC/[39.68 + 1.88 × BMI] × TG/1.03 × 1.31/HDL-C; Female: WC/[36.58 + 1.89 × BMI] × TG/0.81 × 1.52/HDL-C | TG and HDL-C in mmol/L; WC in cm; BMI in kg/m²; sex-specific equation |
| CVAI | Male: −267.93 + 0.68 × age + 0.03 × BMI + 4.00 × WC + 22.00 × log10(TG) − 16.32 × HDL-C; Female: −187.32 + 1.71 × age + 4.23 × BMI + 1.12 × WC + 39.76 × log10(TG) − 11.66 × HDL-C | TG and HDL-C in mmol/L; age in years; WC in cm; BMI in kg/m²; sex-specific equation |
| TCBI | {[TG (mmol/L) × 88.57] × [TC (mmol/L) × 38.67] × body weight (kg)}/1000 | TG and TC entered in mmol/L and converted inside the published formula; weight in kg |
| NHHR | [TC (mmol/L) − HDL-C (mmol/L)]/HDL-C (mmol/L) | TC and HDL-C in mmol/L; the common conversion factor cancels |
| NHHR-WC | NHHR × WC (cm) | NHHR as defined above; WC in cm |

Note: All biochemical inputs are entered in mmol/L. Formulae originally defined with conventional units perform the stated internal conversion.

Supplementary Table 2. Unadjusted ROC performance and sample-specific operating points of 13 indices for identifying IDF-defined MetS.

| **Model** | **Variable** | **AUC** | **95% CI** | **Sample-specific operating point** | **Sensitivity** | **Specificity** | **N** |
| --- | --- | --- | --- | --- | --- | --- | --- |
| Unadjusted | TyG | 0.888 | 0.879–0.896 | 8.730 | 0.848 | 0.811 | 9,660 |
| Unadjusted | TyG-WC | 0.911 | 0.905–0.918 | 777.526 | 0.844 | 0.810 | 9,660 |
| Unadjusted | TyG-WHtR | 0.924 | 0.919–0.930 | 4.655 | 0.907 | 0.804 | 9,660 |
| Unadjusted | CHG | 0.848 | 0.838–0.857 | 5.186 | 0.830 | 0.713 | 9,660 |
| Unadjusted | CHG-WC | 0.893 | 0.885–0.900 | 456.825 | 0.866 | 0.754 | 9,660 |
| Unadjusted | TCBI | 0.850 | 0.840–0.859 | 1906.015 | 0.791 | 0.774 | 9,660 |
| Unadjusted | CMI | 0.897 | 0.890–0.905 | 0.661 | 0.835 | 0.820 | 9,660 |
| Unadjusted | LAP | 0.915 | 0.908–0.921 | 41.520 | 0.855 | 0.829 | 9,660 |
| Unadjusted | NHHR | 0.789 | 0.777–0.800 | 2.955 | 0.801 | 0.646 | 9,660 |
| Unadjusted | NHHR-WC | 0.839 | 0.830–0.849 | 270.543 | 0.799 | 0.721 | 9,660 |
| Unadjusted | METS-IR | 0.905 | 0.898–0.911 | 37.633 | 0.851 | 0.801 | 9,660 |
| Unadjusted | VAI | 0.884 | 0.876–0.892 | 1.774 | 0.850 | 0.777 | 9,660 |
| Unadjusted | CVAI | 0.894 | 0.887–0.901 | 100.340 | 0.881 | 0.766 | 9,660 |

Note: Operating points are sample-specific index values selected using the Youden index and are not clinically validated diagnostic thresholds.

Supplementary Table 3. Model 1 ROC performance and sample-specific probability operating points of 13 indices for identifying IDF-defined MetS.

| **Model** | **Variable** | **AUC** | **95% CI** | **Sample-specific probability operating point** | **Sensitivity** | **Specificity** | **N** |
| --- | --- | --- | --- | --- | --- | --- | --- |
| Model 1 | TyG | 0.894 | 0.886–0.902 | 0.182 | 0.835 | 0.817 | 9,660 |
| Model 1 | TyG-WC | 0.933 | 0.927–0.937 | 0.125 | 0.942 | 0.793 | 9,660 |
| Model 1 | TyG-WHtR | 0.926 | 0.920–0.931 | 0.138 | 0.914 | 0.796 | 9,660 |
| Model 1 | CHG | 0.853 | 0.845–0.863 | 0.136 | 0.848 | 0.696 | 9,660 |
| Model 1 | CHG-WC | 0.915 | 0.908–0.921 | 0.146 | 0.894 | 0.794 | 9,660 |
| Model 1 | TCBI | 0.851 | 0.841–0.860 | 0.159 | 0.830 | 0.724 | 9,660 |
| Model 1 | CMI | 0.903 | 0.896–0.910 | 0.165 | 0.842 | 0.821 | 9,660 |
| Model 1 | LAP | 0.918 | 0.912–0.924 | 0.150 | 0.888 | 0.807 | 9,660 |
| Model 1 | NHHR | 0.815 | 0.805–0.825 | 0.145 | 0.803 | 0.679 | 9,660 |
| Model 1 | NHHR-WC | 0.861 | 0.851–0.869 | 0.140 | 0.835 | 0.726 | 9,660 |
| Model 1 | METS-IR | 0.922 | 0.916–0.928 | 0.131 | 0.903 | 0.789 | 9,660 |
| Model 1 | VAI | 0.895 | 0.887–0.902 | 0.174 | 0.823 | 0.819 | 9,660 |
| Model 1 | CVAI | 0.914 | 0.909–0.920 | 0.150 | 0.922 | 0.793 | 9,660 |

Note: Model 1 includes age and sex. Probability operating points are sample-specific references derived from model-predicted probabilities and are not clinically validated diagnostic thresholds.

Supplementary Table 4. Ranking of AUCs for all 13 indices across 11 prespecified age, sex, and combined age-sex strata under Model 2.

| **Rank** | **20–44 years** | **45–64 years** | **≥65 years** | **Female** | **Female 20–44** | **Female 45–64** | **Female ≥65** | **Male** | **Male 20–44** | **Male 45–64** | **Male ≥65** |
| --- | --- | --- | --- | --- | --- | --- | --- | --- | --- | --- | --- |
| 1 | TyG-WC (0.954) | TyG-WC (0.905) | TyG-WC (0.874) | TyG-WC (0.949) | LAP (0.976) | TyG-WC (0.915) | CHG (0.888) | TyG-WC (0.915) | TyG-WC (0.931) | TyG-WC (0.893) | TyG-WC (0.868) |
| 2 | LAP (0.949) | TyG-WHtR (0.901) | CHG-WC (0.871) | METS-IR (0.949) | TyG-WC (0.972) | TyG-WHtR (0.913) | TyG (0.882) | TyG-WHtR (0.906) | TyG-WHtR (0.923) | TyG-WHtR (0.886) | CHG-WC (0.858) |
| 3 | TyG-WHtR (0.948) | CHG-WC (0.894) | TyG-WHtR (0.855) | LAP (0.948) | CMI (0.971) | METS-IR (0.913) | CHG-WC (0.879) | CHG-WC (0.896) | LAP (0.919) | CHG-WC (0.876) | LAP (0.844) |
| 4 | METS-IR (0.945) | METS-IR (0.889) | LAP (0.847) | CVAI (0.946) | METS-IR (0.970) | CVAI (0.912) | TyG-WC (0.877) | METS-IR (0.893) | METS-IR (0.913) | METS-IR (0.862) | TyG-WHtR (0.841) |
| 5 | CVAI (0.940) | CVAI (0.886) | TyG (0.841) | TyG-WHtR (0.946) | TyG-WHtR (0.967) | CHG-WC (0.910) | TyG-WHtR (0.866) | LAP (0.891) | CHG-WC (0.909) | LAP (0.860) | METS-IR (0.821) |
| 6 | CHG-WC (0.938) | LAP (0.884) | CHG (0.838) | CMI (0.944) | CVAI (0.966) | LAP (0.907) | LAP (0.857) | CVAI (0.882) | CVAI (0.904) | CVAI (0.856) | CVAI (0.818) |
| 7 | CMI (0.934) | TyG (0.864) | METS-IR (0.831) | CHG-WC (0.941) | VAI (0.966) | CMI (0.903) | CMI (0.856) | TyG (0.868) | CMI (0.896) | TyG (0.842) | TyG (0.797) |
| 8 | VAI (0.927) | CMI (0.863) | CVAI (0.815) | NHHR-WC (0.937) | NHHR-WC (0.961) | NHHR-WC (0.899) | VAI (0.852) | CMI (0.864) | TyG (0.889) | CMI (0.827) | CHG (0.794) |
| 9 | TyG (0.927) | CHG (0.858) | NHHR-WC (0.809) | VAI (0.934) | CHG-WC (0.959) | CHG (0.893) | NHHR-WC (0.840) | VAI (0.851) | VAI (0.884) | CHG (0.820) | CMI (0.780) |
| 10 | NHHR-WC (0.915) | VAI (0.854) | CMI (0.809) | TyG (0.930) | TCBI (0.954) | VAI (0.888) | METS-IR (0.832) | NHHR-WC (0.841) | NHHR-WC (0.869) | VAI (0.814) | NHHR-WC (0.778) |
| 11 | CHG (0.899) | NHHR-WC (0.844) | VAI (0.806) | CHG (0.927) | TyG (0.952) | TyG (0.881) | TCBI (0.814) | CHG (0.837) | TCBI (0.853) | NHHR-WC (0.792) | VAI (0.767) |
| 12 | TCBI (0.892) | TCBI (0.807) | TCBI (0.792) | TCBI (0.919) | CHG (0.937) | NHHR (0.855) | CVAI (0.813) | TCBI (0.821) | CHG (0.847) | TCBI (0.782) | TCBI (0.761) |
| 13 | NHHR (0.880) | NHHR (0.795) | NHHR (0.748) | NHHR (0.908) | NHHR (0.929) | TCBI (0.854) | NHHR (0.786) | NHHR (0.788) | NHHR (0.817) | NHHR (0.734) | NHHR (0.710) |

Note: Subgroup AUC rankings are descriptive. Rankings were based on full-precision AUC estimates before rounding; displayed AUCs were rounded to three decimal places. The ≥65-year subgroup, particularly the male and female strata, had relatively small sample sizes; findings in these older-adult strata should be interpreted as exploratory and hypothesis-generating pending external validation.

Supplementary Table 5. Top three indices and reference-profile equivalent index values within each prespecified stratum under Model 2.

| **Group** | **Rank** | **Indicator** | **AUC** | **95% CI** | **Reference-profile equivalent index value** | **Sensitivity** | **Specificity** | **N** |
| --- | --- | --- | --- | --- | --- | --- | --- | --- |
| Male | 1 | TyG-WC | 0.915 | 0.907–0.923 | 823.545 | 0.895 | 0.797 | 4,698 |
| Male | 2 | TyG-WHtR | 0.906 | 0.897–0.914 | 4.858 | 0.851 | 0.811 | 4,698 |
| Male | 3 | CHG-WC | 0.896 | 0.887–0.905 | 483.814 | 0.900 | 0.734 | 4,698 |
| Female | 1 | TyG-WC | 0.949 | 0.942–0.955 | 715.184 | 0.944 | 0.841 | 4,962 |
| Female | 2 | METS-IR | 0.949 | 0.942–0.955 | 36.195 | 0.935 | 0.842 | 4,962 |
| Female | 3 | LAP | 0.948 | 0.940–0.955 | 37.535 | 0.915 | 0.853 | 4,962 |
| 20–44 | 1 | TyG-WC | 0.954 | 0.948–0.960 | 717.363 | 0.951 | 0.847 | 5,415 |
| 20–44 | 2 | LAP | 0.949 | 0.942–0.956 | 44.719 | 0.912 | 0.868 | 5,415 |
| 20–44 | 3 | TyG-WHtR | 0.948 | 0.942–0.954 | 4.507 | 0.930 | 0.847 | 5,415 |
| 45–64 | 1 | TyG-WC | 0.905 | 0.895–0.915 | 723.607 | 0.912 | 0.754 | 3,861 |
| 45–64 | 2 | TyG-WHtR | 0.901 | 0.891–0.911 | 4.648 | 0.879 | 0.781 | 3,861 |
| 45–64 | 3 | CHG-WC | 0.894 | 0.884–0.903 | 432.139 | 0.874 | 0.754 | 3,861 |
| ≥65 | 1 | TyG-WC | 0.874 | 0.839–0.907 | 831.584 | 0.815 | 0.785 | 384 |
| ≥65 | 2 | CHG-WC | 0.871 | 0.836–0.906 | 496.575 | 0.927 | 0.692 | 384 |
| ≥65 | 3 | TyG-WHtR | 0.855 | 0.817–0.890 | 4.948 | 0.863 | 0.685 | 384 |
| Male, 20–44 | 1 | TyG-WC | 0.931 | 0.922–0.941 | 815.969 | 0.927 | 0.810 | 2,605 |
| Male, 20–44 | 2 | TyG-WHtR | 0.923 | 0.913–0.934 | 4.662 | 0.953 | 0.762 | 2,605 |
| Male, 20–44 | 3 | LAP | 0.919 | 0.907–0.930 | 44.940 | 0.941 | 0.771 | 2,605 |
| Male, 45–64 | 1 | TyG-WC | 0.893 | 0.878–0.907 | 819.991 | 0.890 | 0.755 | 1,897 |
| Male, 45–64 | 2 | TyG-WHtR | 0.886 | 0.870–0.901 | 4.877 | 0.865 | 0.784 | 1,897 |
| Male, 45–64 | 3 | CHG-WC | 0.876 | 0.861–0.891 | 497.973 | 0.814 | 0.780 | 1,897 |
| Male, ≥65 | 1 | TyG-WC | 0.868 | 0.809–0.918 | 819.814 | 0.784 | 0.814 | 196 |
| Male, ≥65 | 2 | CHG-WC | 0.858 | 0.798–0.909 | 487.284 | 0.843 | 0.738 | 196 |
| Male, ≥65 | 3 | LAP | 0.844 | 0.781–0.904 | 37.944 | 0.804 | 0.779 | 196 |
| Female, 20–44 | 1 | LAP | 0.976 | 0.966–0.984 | 35.905 | 0.934 | 0.926 | 2,810 |
| Female, 20–44 | 2 | TyG-WC | 0.972 | 0.965–0.979 | 714.472 | 0.949 | 0.920 | 2,810 |
| Female, 20–44 | 3 | CMI | 0.971 | 0.959–0.981 | 0.669 | 0.897 | 0.941 | 2,810 |
| Female, 45–64 | 1 | TyG-WC | 0.915 | 0.902–0.928 | 721.521 | 0.905 | 0.791 | 1,964 |
| Female, 45–64 | 2 | TyG-WHtR | 0.913 | 0.900–0.926 | 4.689 | 0.852 | 0.834 | 1,964 |
| Female, 45–64 | 3 | METS-IR | 0.913 | 0.899–0.926 | 35.792 | 0.860 | 0.824 | 1,964 |
| Female, ≥65 | 1 | CHG | 0.888 | 0.835–0.934 | 5.270 | 0.753 | 0.870 | 188 |
| Female, ≥65 | 2 | TyG | 0.882 | 0.830–0.929 | 8.846 | 0.726 | 0.904 | 188 |
| Female, ≥65 | 3 | CHG-WC | 0.879 | 0.829–0.924 | 452.552 | 0.849 | 0.791 | 188 |

Note: Adjusted ROC analyses were based on model-predicted probabilities. Reference-profile equivalent index values correspond to the Youden-selected probability thresholds, with continuous covariates fixed at their within-stratum medians and categorical covariates at their within-stratum modal levels. These values are not direct univariable ROC cutoffs or clinically validated diagnostic thresholds. AUCs, 95% CIs, reference-profile equivalent index values, sensitivity, and specificity are reported to three decimal places. Rankings were based on full-precision AUC estimates before rounding; displayed AUCs were rounded to three decimal places.

Supplementary Table 6. Overall and nonlinear associations between 13 indices and IDF-defined MetS based on restricted cubic splines.

| **Variable** | **N** | **Knot locations** | **P overall** | **P for nonlinear** |
| --- | --- | --- | --- | --- |
| TyG | 9,660 | 7.583, 8.16, 8.643, 9.584 | <0.001 | <0.001 |
| TyG-WC | 9,660 | 523.966, 652.779, 757.463, 937.811 | <0.001 | <0.001 |
| TyG-WHtR | 9,660 | 3.239, 3.97, 4.577, 5.61 | <0.001 | <0.001 |
| CHG | 9,660 | 4.63, 4.963, 5.211, 5.619 | <0.001 | <0.001 |
| CHG-WC | 9,660 | 318.479, 395.978, 456.959, 553.938 | <0.001 | <0.001 |
| TCBI | 9,660 | 463.138, 1003.352, 1794.204, 5288.284 | <0.001 | <0.001 |
| CMI | 9,660 | 0.142, 0.309, 0.581, 1.686 | <0.001 | <0.001 |
| LAP | 9,660 | 5.6, 17.76, 35.67, 104.063 | <0.001 | <0.001 |
| NHHR | 9,660 | 1.515, 2.389, 3.182, 4.563 | <0.001 | <0.001 |
| NHHR-WC | 9,660 | 110.617, 190.408, 276.677, 433.118 | <0.001 | <0.001 |
| METS-IR | 9,660 | 24.457, 30.964, 36.677, 47.785 | <0.001 | <0.001 |
| VAI | 9,660 | 0.513, 0.996, 1.7, 4.377 | <0.001 | <0.001 |
| CVAI | 9,660 | 0.703, 57.481, 99.223, 159.118 | <0.001 | <0.001 |

Supplementary Table 7. Exposure-specific variance inflation factor diagnostics for Model 2.

| **Exposure** | **Covariate** | **VIF** | **R2** | **N** |
| --- | --- | --- | --- | --- |
| CVAI | Gender - Female | 2.768 | 0.639 | 9,660 |
| CHG-WC | Gender - Female | 2.760 | 0.638 | 9,660 |
| TyG-WC | Gender - Female | 2.680 | 0.627 | 9,660 |
| NHHR-WC | Gender - Female | 2.596 | 0.615 | 9,660 |
| CHG | Gender - Female | 2.533 | 0.605 | 9,660 |
| NHHR | Gender - Female | 2.505 | 0.601 | 9,660 |
| METS-IR | Gender - Female | 2.489 | 0.598 | 9,660 |
| TyG-WHtR | Gender - Female | 2.451 | 0.592 | 9,660 |
| TyG | Gender - Female | 2.431 | 0.589 | 9,660 |
| LAP | Gender - Female | 2.383 | 0.580 | 9,660 |
| CMI | Gender - Female | 2.379 | 0.580 | 9,660 |
| CVAI | CVAI | 2.375 | 0.579 | 9,660 |
| TCBI | Gender - Female | 2.370 | 0.578 | 9,660 |
| VAI | Gender - Female | 2.353 | 0.575 | 9,660 |
| TyG-WHtR | Cr | 2.235 | 0.553 | 9,660 |
| CHG | Cr | 2.234 | 0.552 | 9,660 |
| CHG-WC | Cr | 2.232 | 0.552 | 9,660 |
| TyG-WC | Cr | 2.226 | 0.551 | 9,660 |
| LAP | Cr | 2.224 | 0.550 | 9,660 |
| CVAI | Cr | 2.218 | 0.549 | 9,660 |
| VAI | Cr | 2.217 | 0.549 | 9,660 |
| CMI | Cr | 2.216 | 0.549 | 9,660 |
| METS-IR | Cr | 2.215 | 0.549 | 9,660 |
| NHHR-WC | Cr | 2.215 | 0.549 | 9,660 |
| TyG | Cr | 2.214 | 0.548 | 9,660 |
| TCBI | Cr | 2.211 | 0.548 | 9,660 |
| NHHR | Cr | 2.207 | 0.547 | 9,660 |
| CVAI | UA | 2.063 | 0.515 | 9,660 |
| TyG-WC | UA | 2.037 | 0.509 | 9,660 |
| METS-IR | UA | 2.033 | 0.508 | 9,660 |
| CHG-WC | CHG-WC | 2.032 | 0.508 | 9,660 |
| TyG-WHtR | UA | 2.028 | 0.507 | 9,660 |
| CHG-WC | UA | 2.001 | 0.500 | 9,660 |
| NHHR-WC | NHHR-WC | 1.999 | 0.500 | 9,660 |
| NHHR | NHHR | 1.953 | 0.488 | 9,660 |
| NHHR-WC | UA | 1.915 | 0.478 | 9,660 |
| TyG-WC | TyG-WC | 1.897 | 0.473 | 9,660 |
| TyG | UA | 1.895 | 0.472 | 9,660 |
| NHHR | UA | 1.857 | 0.461 | 9,660 |
| LAP | UA | 1.849 | 0.459 | 9,660 |
| CHG | CHG | 1.848 | 0.459 | 9,660 |
| CHG | UA | 1.846 | 0.458 | 9,660 |
| VAI | UA | 1.824 | 0.452 | 9,660 |
| CMI | UA | 1.817 | 0.450 | 9,660 |
| TCBI | UA | 1.803 | 0.445 | 9,660 |
| TyG-WHtR | TyG-WHtR | 1.644 | 0.392 | 9,660 |
| NHHR | LDL-C | 1.643 | 0.392 | 9,660 |
| CVAI | Age | 1.610 | 0.379 | 9,660 |
| CHG | LDL-C | 1.500 | 0.333 | 9,660 |
| METS-IR | METS-IR | 1.498 | 0.332 | 9,660 |
| NHHR-WC | LDL-C | 1.480 | 0.324 | 9,660 |
| TyG | TyG | 1.421 | 0.296 | 9,660 |
| TyG-WHtR | Age | 1.318 | 0.241 | 9,660 |
| TyG | BUN | 1.260 | 0.206 | 9,660 |
| TyG-WC | BUN | 1.258 | 0.205 | 9,660 |
| TyG-WHtR | BUN | 1.258 | 0.205 | 9,660 |
| CVAI | BUN | 1.257 | 0.205 | 9,660 |
| METS-IR | BUN | 1.256 | 0.204 | 9,660 |
| NHHR | BUN | 1.255 | 0.203 | 9,660 |
| NHHR-WC | BUN | 1.255 | 0.203 | 9,660 |
| CHG-WC | BUN | 1.254 | 0.202 | 9,660 |
| CHG | BUN | 1.251 | 0.201 | 9,660 |
| CMI | BUN | 1.251 | 0.200 | 9,660 |
| LAP | BUN | 1.251 | 0.200 | 9,660 |
| TCBI | BUN | 1.251 | 0.201 | 9,660 |
| VAI | BUN | 1.251 | 0.200 | 9,660 |
| TyG-WC | Age | 1.235 | 0.190 | 9,660 |
| CHG-WC | Age | 1.234 | 0.190 | 9,660 |
| TyG | LDL-C | 1.185 | 0.156 | 9,660 |
| CHG-WC | LDL-C | 1.180 | 0.153 | 9,660 |
| TyG | Age | 1.179 | 0.152 | 9,660 |
| CHG | Age | 1.177 | 0.151 | 9,660 |
| METS-IR | Age | 1.162 | 0.139 | 9,660 |
| LAP | LAP | 1.141 | 0.123 | 9,660 |
| TCBI | LDL-C | 1.133 | 0.118 | 9,660 |
| TyG-WHtR | LDL-C | 1.133 | 0.117 | 9,660 |
| NHHR-WC | Age | 1.129 | 0.114 | 9,660 |
| TyG-WC | LDL-C | 1.129 | 0.114 | 9,660 |
| LAP | Age | 1.121 | 0.108 | 9,660 |
| NHHR | Age | 1.119 | 0.106 | 9,660 |
| VAI | Age | 1.119 | 0.106 | 9,660 |
| CMI | Age | 1.118 | 0.105 | 9,660 |
| TCBI | Age | 1.117 | 0.105 | 9,660 |
| LAP | LDL-C | 1.115 | 0.103 | 9,660 |
| METS-IR | TBIL | 1.112 | 0.100 | 9,660 |
| NHHR | TBIL | 1.112 | 0.101 | 9,660 |
| NHHR-WC | TBIL | 1.112 | 0.100 | 9,660 |
| CHG | TBIL | 1.109 | 0.098 | 9,660 |
| TyG-WHtR | TBIL | 1.109 | 0.098 | 9,660 |
| CHG-WC | TBIL | 1.106 | 0.096 | 9,660 |
| TyG-WC | TBIL | 1.106 | 0.096 | 9,660 |
| CVAI | TBIL | 1.105 | 0.095 | 9,660 |
| TyG | TBIL | 1.105 | 0.095 | 9,660 |
| CVAI | LDL-C | 1.102 | 0.092 | 9,660 |
| LAP | TBIL | 1.100 | 0.091 | 9,660 |
| VAI | LDL-C | 1.100 | 0.091 | 9,660 |
| CMI | LDL-C | 1.098 | 0.090 | 9,660 |
| CMI | TBIL | 1.098 | 0.089 | 9,660 |
| TCBI | TBIL | 1.098 | 0.089 | 9,660 |
| VAI | TBIL | 1.098 | 0.090 | 9,660 |
| METS-IR | LDL-C | 1.092 | 0.084 | 9,660 |
| TCBI | TCBI | 1.089 | 0.082 | 9,660 |
| CMI | CMI | 1.075 | 0.069 | 9,660 |
| VAI | VAI | 1.058 | 0.055 | 9,660 |

Note: VIFs are reported for all terms in each exposure-specific Model 2, including the exposure term and adjustment covariates.

**Supplementary Table 8. Main and winsorized Model 2 logistic associations under two parallel standardization approaches**

| **Indicator** | **Main SD** | **Winsorized SD** | **Main skewness** | **Winsorized skewness** | **Main OR (95% CI)** | **Main P** | **Winsorized OR (95% CI), self-SD** | **Winsorized OR (95% CI), fixed main-SD** | **Winsorized P** | **Excess-OR change, self-SD (%)** | **Excess-OR change, fixed main-SD (%)** |
| --- | --- | --- | --- | --- | --- | --- | --- | --- | --- | --- | --- |
| CMI | 1.295 | 0.592 | 30.571 | 3.189 | 19.437 (16.039–23.554) | <0.001 | 4.818 (4.380–5.299) | 31.215 (25.342–38.448) | <0.001 | -79.3 | 63.9 |
| TyG-WC | 130.195 | 126.573 | 0.542 | 0.386 | 16.332 (14.167–18.828) | <0.001 | 17.181 (14.885–19.832) | 18.638 (16.081–21.602) | <0.001 | 5.5 | 15.0 |
| LAP | 61.941 | 36.411 | 15.374 | 2.813 | 15.482 (13.152–18.224) | <0.001 | 6.477 (5.830–7.195) | 24.001 (20.069–28.704) | <0.001 | -62.2 | 58.8 |
| CVAI | 49.137 | 48.358 | 0.170 | 0.092 | 13.768 (11.976–15.828) | <0.001 | 14.279 (12.420–16.417) | 14.904 (12.934–17.174) | <0.001 | 4.0 | 8.9 |
| CHG-WC | 74.289 | 72.308 | 0.439 | 0.282 | 13.062 (11.435–14.920) | <0.001 | 13.522 (11.836–15.447) | 14.522 (12.665–16.651) | <0.001 | 3.8 | 12.1 |
| TyG-WHtR | 0.738 | 0.717 | 0.551 | 0.399 | 12.006 (10.569–13.638) | <0.001 | 12.505 (10.998–14.218) | 13.469 (11.801–15.372) | <0.001 | 4.5 | 13.3 |
| TCBI | 5503.425 | 1915.716 | 28.191 | 3.552 | 11.971 (9.545–15.014) | <0.001 | 4.045 (3.668–4.461) | 55.414 (41.844–73.385) | <0.001 | -72.2 | 396.0 |
| VAI | 2.934 | 1.467 | 25.084 | 3.034 | 11.615 (9.905–13.619) | <0.001 | 4.104 (3.768–4.469) | 16.847 (14.205–19.980) | <0.001 | -70.8 | 49.3 |
| METS-IR | 7.370 | 7.102 | 0.845 | 0.615 | 9.470 (8.441–10.624) | <0.001 | 9.470 (8.448–10.616) | 10.308 (9.156–11.605) | <0.001 | 0.0 | 9.9 |
| NHHR-WC | 110.062 | 101.477 | 2.976 | 0.860 | 9.280 (8.203–10.498) | <0.001 | 8.336 (7.422–9.363) | 9.975 (8.794–11.314) | <0.001 | -11.4 | 8.4 |
| CHG | 0.325 | 0.306 | 0.920 | 0.428 | 6.961 (6.218–7.792) | <0.001 | 7.056 (6.321–7.877) | 7.929 (7.056–8.910) | <0.001 | 1.6 | 16.2 |
| TyG | 0.645 | 0.613 | 1.067 | 0.677 | 5.932 (5.376–6.546) | <0.001 | 5.917 (5.376–6.512) | 6.489 (5.866–7.178) | <0.001 | -0.3 | 11.3 |
| NHHR | 1.032 | 0.953 | 2.603 | 0.704 | 4.891 (4.415–5.419) | <0.001 | 4.525 (4.108–4.985) | 5.127 (4.617–5.692) | <0.001 | -9.4 | 6.1 |

Note: Model 2 adjusted for age, sex, LDL-C, TBIL, BUN, Cr, and UA. Skewness was calculated descriptively for each main-analysis index and its recalculated winsorized counterpart using the adjusted Fisher-Pearson sample skewness; no covariate adjustment was applied to this distributional diagnostic. The self-SD and fixed main-SD approaches were treated as two parallel sensitivity analyses of the same winsorized dataset. Under self-SD, recalculated indices were standardized using the means and SDs from the winsorized data; under fixed main-SD, the same recalculated indices were standardized using the corresponding means and SDs from the main-analysis data. Because self-SD and fixed main-SD standardization are linear rescalings of the same winsorized indices fitted to the same Model 2 data, their Wald P values are identical; therefore, one winsorized P value is reported. Excess-OR change = 100 × [OR (winsorized) − OR (main)]/[OR (main) − 1]; positive values indicate amplification and negative values indicate attenuation relative to the main analysis.

**Supplementary Table 9. Main versus winsorized overall Model 2 ROC performance**

| **Indicator** | **Main AUC** | **Main 95% CI** | **Main sensitivity** | **Main specificity** | **Winsorized AUC** | **Winsorized 95% CI** | **Winsorized sensitivity** | **Winsorized specificity** | **ΔAUC** |
| --- | --- | --- | --- | --- | --- | --- | --- | --- | --- |
| TyG-WC | 0.934 | 0.929–0.939 | 0.930 | 0.810 | 0.935 | 0.930–0.939 | 0.926 | 0.814 | +0.001 |
| TyG-WHtR | 0.928 | 0.922–0.933 | 0.893 | 0.823 | 0.928 | 0.923–0.934 | 0.902 | 0.816 | 0.000 |
| METS-IR | 0.923 | 0.917–0.929 | 0.900 | 0.794 | 0.923 | 0.917–0.929 | 0.881 | 0.812 | 0.000 |
| CHG-WC | 0.921 | 0.915–0.926 | 0.922 | 0.777 | 0.921 | 0.915–0.926 | 0.928 | 0.768 | 0.000 |
| LAP | 0.920 | 0.914–0.926 | 0.876 | 0.814 | 0.922 | 0.917–0.928 | 0.885 | 0.817 | +0.002 |
| CVAI | 0.915 | 0.909–0.920 | 0.938 | 0.775 | 0.914 | 0.909–0.920 | 0.944 | 0.769 | -0.001 |
| CMI | 0.903 | 0.895–0.910 | 0.861 | 0.796 | 0.906 | 0.899–0.912 | 0.873 | 0.793 | +0.003 |
| TyG | 0.902 | 0.895–0.909 | 0.847 | 0.822 | 0.903 | 0.896–0.910 | 0.854 | 0.816 | +0.001 |
| VAI | 0.896 | 0.889–0.903 | 0.837 | 0.800 | 0.900 | 0.892–0.906 | 0.828 | 0.819 | +0.004 |
| NHHR-WC | 0.890 | 0.883–0.898 | 0.856 | 0.770 | 0.890 | 0.882–0.898 | 0.855 | 0.770 | 0.000 |
| CHG | 0.885 | 0.877–0.893 | 0.839 | 0.774 | 0.885 | 0.877–0.893 | 0.817 | 0.790 | 0.000 |
| TCBI | 0.863 | 0.855–0.871 | 0.842 | 0.727 | 0.881 | 0.873–0.889 | 0.835 | 0.768 | +0.018 |
| NHHR | 0.852 | 0.843–0.861 | 0.875 | 0.674 | 0.851 | 0.842–0.860 | 0.873 | 0.672 | -0.001 |

Note: N = 9,660 for every index in both analyses. Model 2 adjusted for age, sex, LDL-C, TBIL, BUN, Cr, and UA. For the sensitivity analysis, selected continuous inputs used to construct the indices were winsorized at the 1st and 99th percentiles, the indices were recalculated, and the original IDF-defined MetS outcome and Model 2 covariates were retained. ΔAUC = winsorized AUC − main AUC.

**Supplementary Table 10. Distributional effects of component-specific winsorization on TCBI and its Model 2 ROC discrimination**

| **Scenario** | **Component(s)** | **Component SD, main** | **Component SD, winsorized** | **TCBI SD** | **TCBI values changed, %** | **Model 2 TCBI AUC (95% CI)** | **ΔAUC** |
| --- | --- | --- | --- | --- | --- | --- | --- |
| Main analysis | None | — | — | 5503.425 | 0.000 | 0.863 (0.855–0.871) | Reference |
| TG winsorized only | TG | 1.780 | 1.045 | 2057.786 | 1.884 | 0.880 (0.872–0.888) | +0.017 |
| TC winsorized only | TC | 1.078 | 1.013 | 3864.002 | 1.988 | 0.872 (0.864–0.881) | +0.009 |
| Body weight winsorized only | Body weight | 12.881 | 12.591 | 5500.343 | 1.957 | 0.863 (0.854–0.872) | 0.000 |
| TG + TC + body weight winsorized | TG + TC + body weight | TG 1.780 TC 1.078 Weight 12.881 | TG 1.045 TC 1.013 Weight 12.591 | 1915.716 | 5.404 | 0.881 (0.873–0.889) | +0.018 |

Note: N = 9,660. TCBI was recalculated after winsorizing TG, TC, body weight, or all three components at the 1st and 99th percentiles. Model 2 covariates and the original IDF-defined MetS outcome were unchanged. ΔAUC is relative to the main-analysis TCBI AUC.

**Supplementary Figure 1. Unadjusted ROC curves for 13 indices identifying IDF-defined MetS.**

**Supplementary Figure 2. Model 1 ROC curves for 13 indices identifying IDF-defined MetS.**

**Supplementary Figure 3. Model 2 ROC curves across 11 prespecified age, sex, and age-by-sex strata. Subgroup AUC rankings are descriptive.**

**Supplementary Figure 4. Model 2 logistic associations in the main analysis and two winsorized standardization approaches. Odds ratios and 95% CIs are reported per 1 SD under the indicated scaling.**
